# Multicontrast investigation of *in vivo* wildtype zebrafish in three development stages using polarization-sensitive optical coherence tomography

**DOI:** 10.1117/1.JBO.27.1.016001

**Published:** 2022-01-21

**Authors:** Antonia Lichtenegger, Pradipta Mukherjee, Junya Tamaoki, Lixuan Bian, Lida Zhu, Ibrahim Abd El-Sadek, Shuichi Makita, Konrad Leskovar, Makoto Kobayashi, Bernhard Baumann, Yoshiaki Yasuno

**Affiliations:** a Medical University of Vienna, Center for Medical Physics and Biomedical Engineering, Vienna, Austria; b University of Tsukuba, Computational Optics Group, Tsukuba, Japan; c University of Tsukuba, Faculty of Medicine, Department of Molecular and Developmental Biology, Tsukuba, Japan; d Damietta University, Faculty of Science, Department of Physics, Damietta, Egypt

**Keywords:** Jones matrix OCT, polarization-sensitive, zebrafish, non-destructive

## Abstract

**Significance:**

The scattering and polarization characteristics of various organs of *in vivo* wildtype zebrafish in three development stages were investigated using a non-destructive and label-free approach. The presented results showed a promising first step for the usability of Jones-matrix optical coherence tomography (JM-OCT) in zebrafish-based research.

**Aim:**

We aim to visualize and quantify the scatter and polarization signatures of various zebrafish organs for larvae, juvenile, and young adult animals *in vivo* in a non-invasive and label-free way.

**Approach:**

A custom-built polarization-sensitive JM-OCT setup in combination with a motorized translation stage was utilized to investigate live zebrafish. Depth-resolved scattering (intensity and attenuation coefficient) and polarization (birefringence and degree of polarization uniformity) properties were analyzed. OCT angiography (OCT-A) was utilized to investigate the vasculature label-free and non-destructively.

**Results:**

The scatter and polarization signatures of the zebrafish organs such as the eye, gills, and muscles were investigated. The attenuation coefficient and birefringence changes between 1- and 2-month-old animals were evaluated in selected organs. OCT-A revealed the vasculature of *in vivo* larvae and juvenile zebrafish in a label-free manner.

**Conclusions:**

JM-OCT offers a rapid, label-free, non-invasive, tissue specific, and three-dimensional imaging tool to investigate *in vivo* processes in zebrafish in various development stages.

## 1 Introduction

In biomedical research, animal models are essential to understanding the pathogenesis of human diseases on a molecular and cellular level and to introduce new therapies. Since the development of important genetic techniques, such as mutagenesis, in the 1980s, the zebrafish has been established as an important animal model in preclinical research.^1^ Zebrafish possess several advantages over traditionally used rodent models such as mice. They are rather easy to handle, and their small size contributes to low costs per animal.^1^ Zebrafish are highly fecund and provide a large number of offspring per clutch.^2^ Importantly, humans and zebrafish share a high level of genome structure.^3^ Furthermore, forward and reverse genetic approaches are well established in zebrafish, making it an attractive model for studying a variety of human diseases.^2^ The transparency of the zebrafish larvae enables an unprecedented direct analysis of pathologic processes *in vivo*, ultimately leading to the possibility of highly efficient studies.^4^

Most investigations are conducted in larvae; however, for some, it is beneficial to examine zebrafish over multiple development stages.^5^ Especially in juvenile and adult zebrafish, conventionally used imaging techniques, such as white-light or fluorescence microscopy, are limited to the skin and subdermal structures.^6^ Other techniques such as magnetic resonance imaging^7^ or computed tomography^8^ are cost intense, limited in their resolution capabilities, complex and until now not feasible for high throughput studies. One way to overcome the depth limitation was the development of transparent mutant zebrafish; however this procedure introduces additional working steps and complications.^6^ To investigate non-transparent zebrafish at various development stages, a real-time, three-dimensional (3D), and non-invasive imaging tool is needed.

Optical coherence tomography (OCT) is a low-coherence light interference-based optical imaging technique that can provide rapid, 3D, and non-destructive images in a label-free manner. OCT offers micrometer resolution over a couple of millimeters. The morphology of the tissue is assessed by analyzing the intrinsic contrast, based on microscopic refractive index changes.^9^ Furthermore, OCT angiography provides a label-free way to investigate the *in vivo* blood flow.^10^ In recent decades, OCT has shown to be a useful tool for many microscopy-related studies.^9^ In the field of zebrafish-based research, the technique has either been used for larva imaging^11^^,^^12^ or in adult animals to investigate the brain^13^^,^^14^ and eye.^15^^,^^16^ Most studies presented so far were conducted with conventional intensity-based OCT setups.^11^^–^^16^

Polarization-sensitive OCT (PS-OCT) provides tissue specific contrast by analyzing the polarization states of the backscattered and reflected light.^17^ The birefringence and depolarization properties of tissue can be measured and quantified label-free and non-destructively. Birefringence is a marker for tissue containing highly fibrous structures, such as muscles and fibrosis.^18^ Depolarization is found in samples that induce multiple scattering or scattering on non-spherical particles such as melanin pigments.^19^ In 2020, Yang et al.^20^ presented PS-OCT results obtained in adult zebrafish that were focused on analyzing the tail musculature based on accumulative polarization measurements. Jones-matrix OCT (JM-OCT) is a specific PS-OCT implementation that is characterized by its ability to measure the 3D distribution of the Jones matrix of the sample. By processing the Jones matrixes, depth-localized phase retardation, which is proportional to birefringence, and local randomness of polarization can be obtained (degree of polarization uniformity; DOPU).^17^^,^^18^

In this work, we utilize a JM-OCT prototype in combination with a motorized translation stage to perform large field-of-view, live wildtype zebrafish investigations in three development stages. The scatter and polarization characteristics of these animals were investigated in a non-destructive manner, and changes over time were analyzed. This work shows the potential of JM-OCT as a non-invasive and label-free tool for *in vivo* zebrafish-based research.

## 2 Methods

### 2.1 Zebrafish Imaging

AB (wild-type) zebrafish at the age of 8-days (N=2), 1-month (N=2), and 2-months (N=2) postfertilization were utilized in this study. The animals were anesthetized using tricaine (3-aminobenzoic acid ethyl ester methane-sulfonate, 0.16  mg/ml) and placed in a water filled Petri dish for imaging. In addition, the 8-day-old animals were embedded in low-melting-point agarose gel (1.5%) for alignment purposes. All animal experiments were performed in accordance with the animal study guidelines of the University of Tsukuba.

### 2.2 Jones-Matrix Optical Coherence Tomography Prototype

A custom-built JM-OCT setup was utilized to investigate the zebrafish. Details of the setup can be found elsewhere.^21^ The JM-OCT prototype was based on a passive-polarization-delay-based PS-OCT scheme. A swept source laser with a central wavelength of 1310 nm was used for imaging. The A-scan rate was 50 kHz, and a system sensitivity of 104 dB was measured with a probe beam power of 11 mW. The axial resolution in tissue was 14  μm with a depth pixel separation of 7.24  μm. The depth range in air was 2.9 mm. For imaging, two alternative scan lenses were utilized, providing lateral resolutions of 18.1  μm (low-resolution LSM03 scanning lens, Thorlabs) and 8.9  μm (high-resolution LSM02 scanning lens, Thorlabs), respectively. The corresponding field of view (FoV) varied from 1  mm×1  mm up to 6  mm×6  mm, respectively.

Large FoV images were acquired with the JM-OCT prototype in combination with a motorized translation stage moving in the $\overset{\to }{x}$ and $\overset{\to }{y}$ directions. The sample was placed on the stage, which was able to scan large field-of-views up to several square centimeters.^22^ The OCT data were acquired using an overlap of 5% between volumes. After postprocessing, the volumes were stitched together using the pairwise stitching plugin of Fiji.^23^

### 2.3 Data Evaluation

To generate OCT intensity data, the absolute-squared intensities of the four Jones matrix entries, corresponding to the four polarization channels, were averaged. The attenuation coefficients were calculated using the depth-resolved method devised by Vermeer et al.^24^ The attenuation represents the scattering and absorption property of the investigated tissue. The depth-resolved birefringence data, also known as the local retardation, were obtained by a local Jones matrix analysis in combination with a maximum *a posteriori* birefringence estimator.^25^^,^^26^ Further, the DOPU was analyzed.^27^ OCT angiography (OCT-A) images were obtained by a complex correlation-based OCT-A approach utilizing four repeated frames.^28^

To quantify the scattering and polarization signatures in 1- and 2-month-old zebrafish organs, a segmentation assistant software (ITK-Snap^29^) was used to manually segment the anatomical features, namely the spinal cord, gills, skeletal muscles, and skin, intensity B-scan-wise. The resulting binary masks were applied to the original volumetric PS-OCT data (attenuation coefficient and birefringence) and used to analyze the distribution of these values. Box plots were created in MATLAB (MATLAB, R2021a, MathWorks), showing the median values (red line), 25% to 75% percentiles (box), maximum and minimum values (horizontal bars), and outliers (red crosses) of the data.

## 3 Results

### 3.1 Eight-Day-Old Zebrafish Imaging

**Fig. 1:**
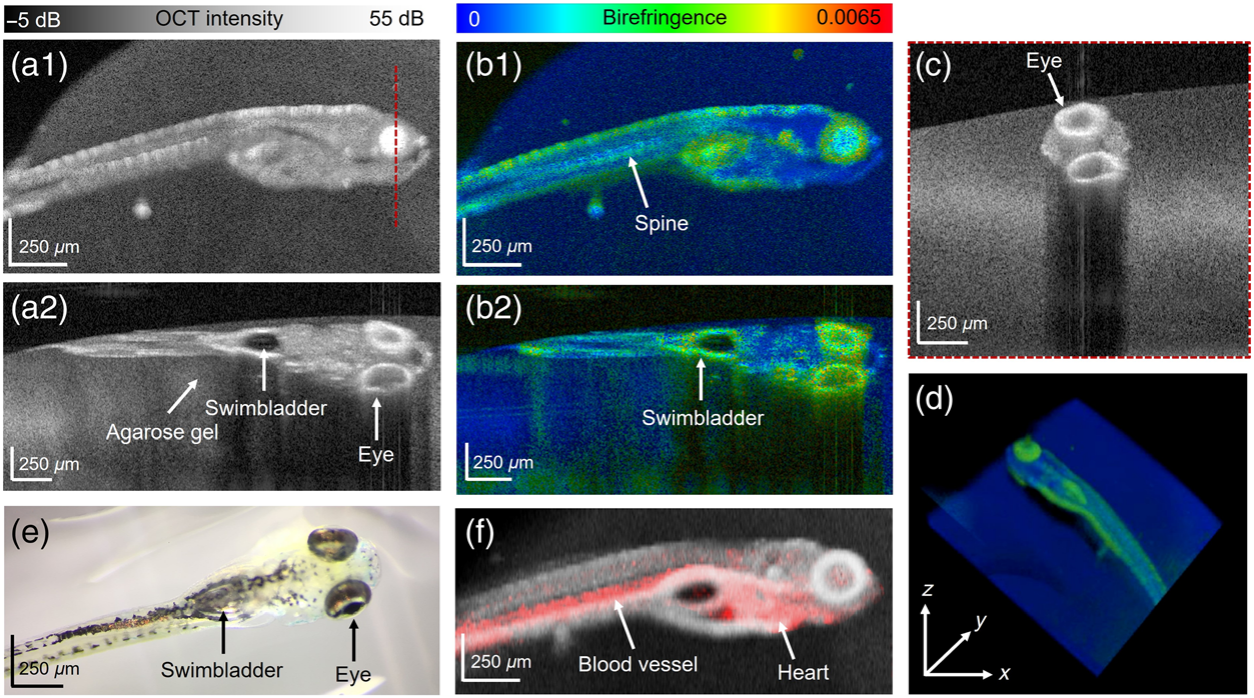
Imaging of 8-day-old zebrafish: (a1) scattering-based *en face* image; (a2) Sagittal B-scan image; (b1) birefringent *en face* image; (b2) birefringent sagittal tomogram; (c) coronal scatter intensity-based cross-section at position indicated in (a1) by a red dashed line; (d) volume rendering of the birefringence data; (e) white-light microscope image; and (f) composition image of the OCT scatter intensity (gray) and angiography (red) data.

The 8-day-old zebrafish were imaged live with the JM-OCT setup in combination with the high-resolution scanning lens. The agarose gel around the larvae exhibited low scattering [see Figs. 1(a2) and 1(c)], respectively. Anatomical features such as the eye and swimbladder wall were identified in the scattering-based images [Figs. 1(a2) and 1(c)] and compared with the white-light stereomicroscope (Olympus, SZX16) image [Fig. 1(e)]. The eye, swimbladder wall, and muscles in the trunk were identified as regions of increased birefringence [see Figs. 1(b1) and 1(b2), respectively]. Additionally, a 3D rendering of the birefringence data is shown in Fig. 1(d). Furthermore, the OCT-A results (red), overlayed with the scattering intensity-data (gray) in Fig. 1(f), revealed the heart region and the large blood vessel in the tail.

**Fig. 2:**
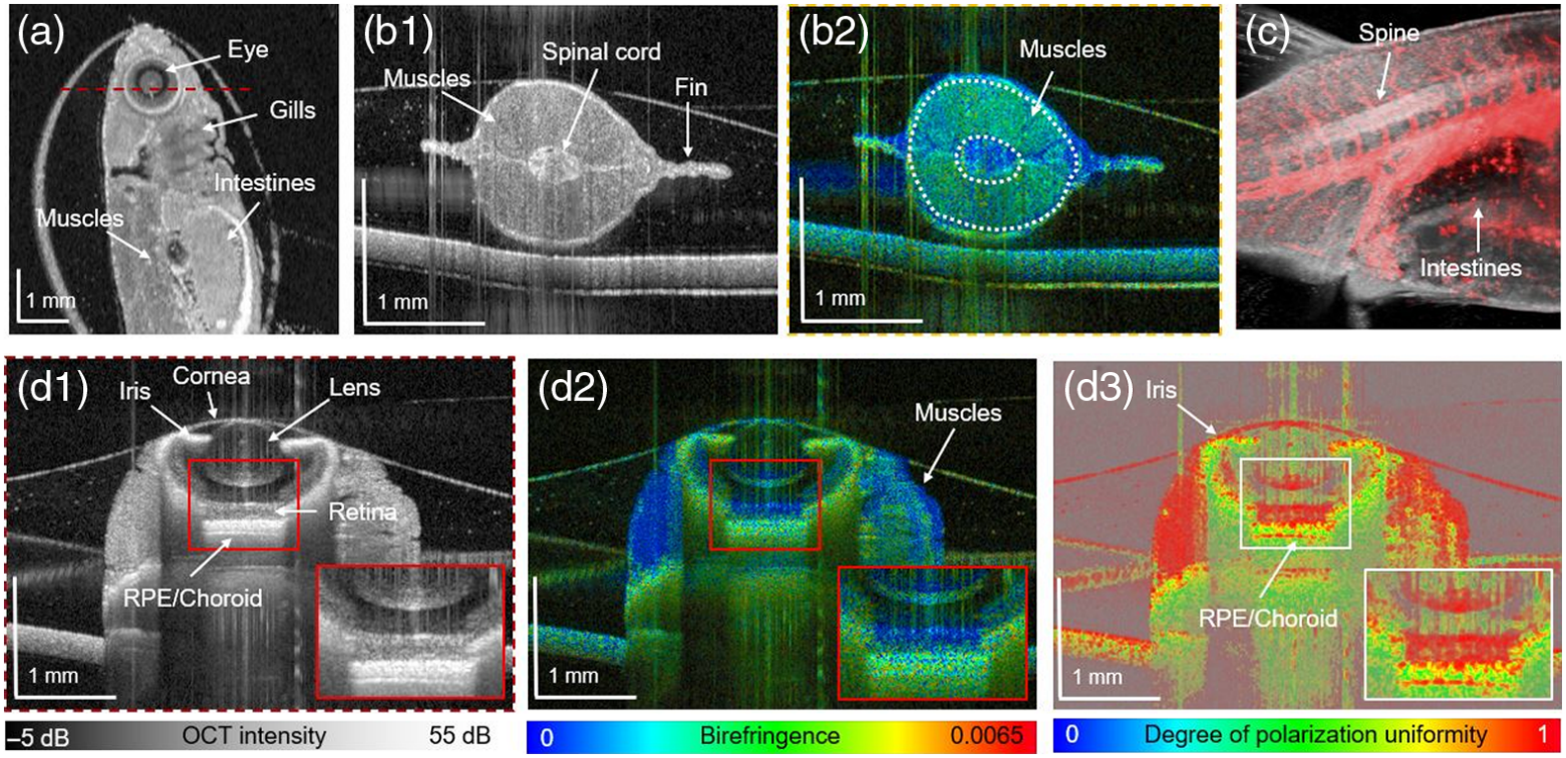
Imaging of 1-month-old zebrafish: (a) *En face* scattering-based image; (b1) transversal B-scan; (b2) birefringence tomogram; (c) composition image of the OCT scatter-intensity (gray) and angiography (red) data; (d1) transversal tomogram at the indicated position by red dashed lines in (a) with a zoom-in; (d2) birefringent-based B-scan; and (d3) DOPU tomogram (RPE, retinal pigment epithelium).

### 3.2 Imaging of 1 and 2-Month-Old Zebrafish

Figure 2 summarizes the 1-month-old zebrafish results investigated with the JM-OCT prototype in combination with the low-resolution scanning lens. With a FoV of 6  mm×6  mm, a large portion of the zebrafish body was imaged [Fig. 2(a)]. The muscles surrounding the spinal cord and fins of the animal were visualized [see Figs. 2(b1) and 2(b2), respectively]. The muscles showed increased birefringence values [Fig. 2(b2)]. The OCT-A data (red) in the fish tail [Fig. 2(c)] revealed the larger and smaller blood vessels supplying the organs and muscles.

Important anatomical features of the eye such as the cornea, iris, lens, retina, and retinal pigment epithelium (RPE)/choroid complex were identified in the scattering-based image [Fig. 2(d1)]. The DOPU image revealed a strong depolarization of the iris and RPE/choroid complex [Fig. 2(d3)]. Furthermore, the cornea and adjacent muscles to the eye showed a moderate birefringence [Fig. 2(d2)]. Tailing artifacts are visible beneath the densely pigmented structures [see Figs. 2(d1)–2(d3)]. The vertical lines in the cross-sectional images in Fig. 2 are artifacts caused by specular reflections.

**Fig. 3:**
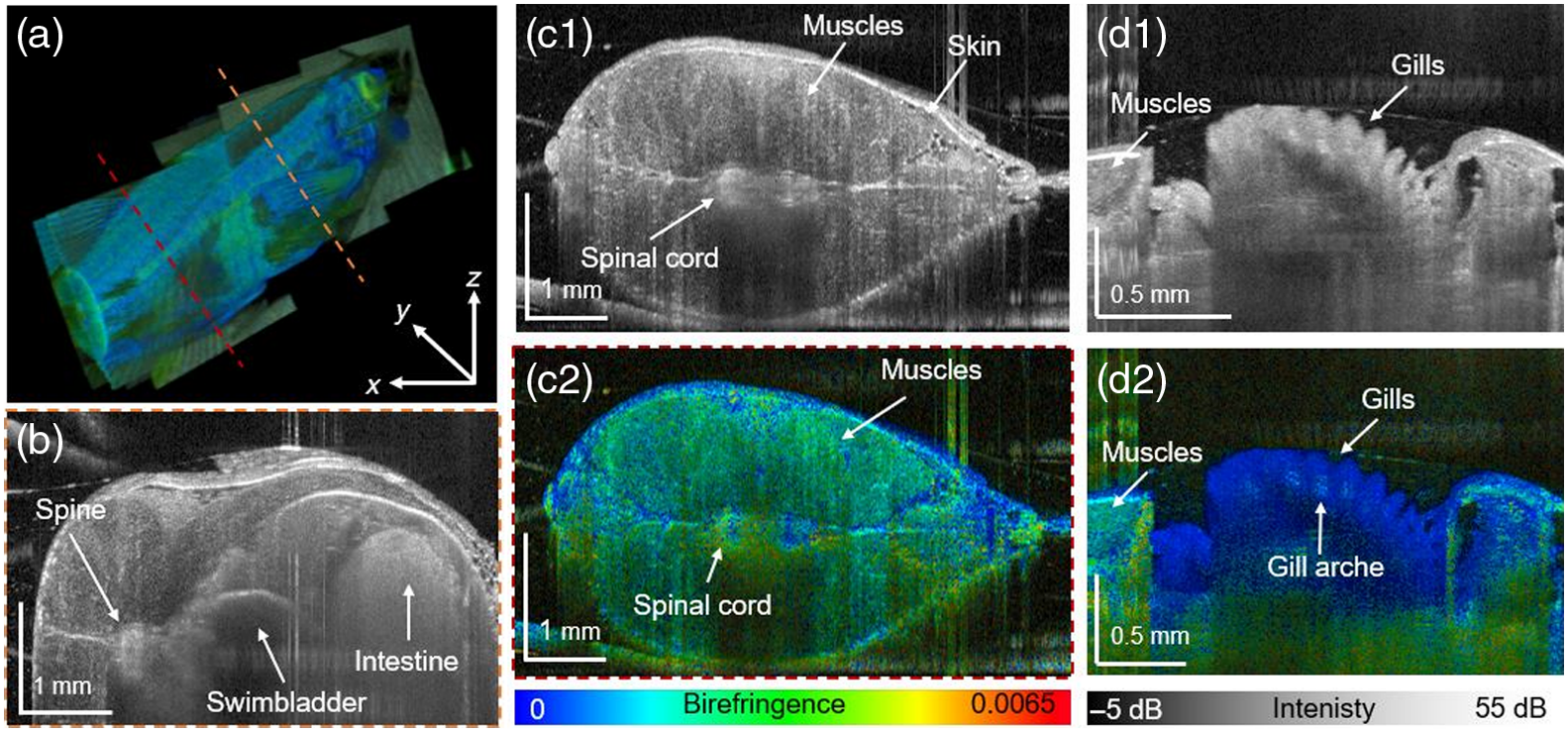
Imaging of 2-month-old zebrafish: (a) 3D-rendering of the birefringence data; (b) transversal scattering-based tomogram at the indicated position by an orange dashed line in (a); (c1) transversal image in the tail region; (c2) birefringence image at the indicated position by a red dashed line; (d1) scattering-based B-scan image of the gills; and (d2) birefringence B-scan (Video 1, MP4, 8.5 MB [URL: https://doi.org/10.1117/1.JBO.27.1.016001.1] and Video 2, MP4, 14 MB [URL: https://doi.org/10.1117/1.JBO.27.1.016001.2]).

The results obtained in the 2-month-old zebrafish are presented in Fig. 3. Three consecutive volumes were stitched together to generate the 3D rendering in Fig. 3(a). Fly-through videos of the scatter intensity (Video 1) and birefringence (Video 2) data can be found in Fig. 3. The cross-sectional OCT image [Fig. 3(b)] in the abdomen region revealed the scattering property of the skin, fin, spinal cord, intestines, and swimbladder. As observed in the 1-month-old fish, the muscles showed an increased birefringence [see Figs. 3(c1) and 3(c2), respectively]. Further, the spinal cord showed up as a region of high birefringence, indicated in Fig. 3(c2). Figures 3(d1) and 3(d2) present cross-sectional images of the gills in a 2-month-old zebrafish in which the operculum, the boney plate protecting the gills, was abnormally absent. The gills exhibited low scattering and birefringence, with some high values found in the cartilage rich arches [Fig. 3(d2)].

### 3.3 Quantitative Scattering and Polarization Analysis

**Fig. 4:**
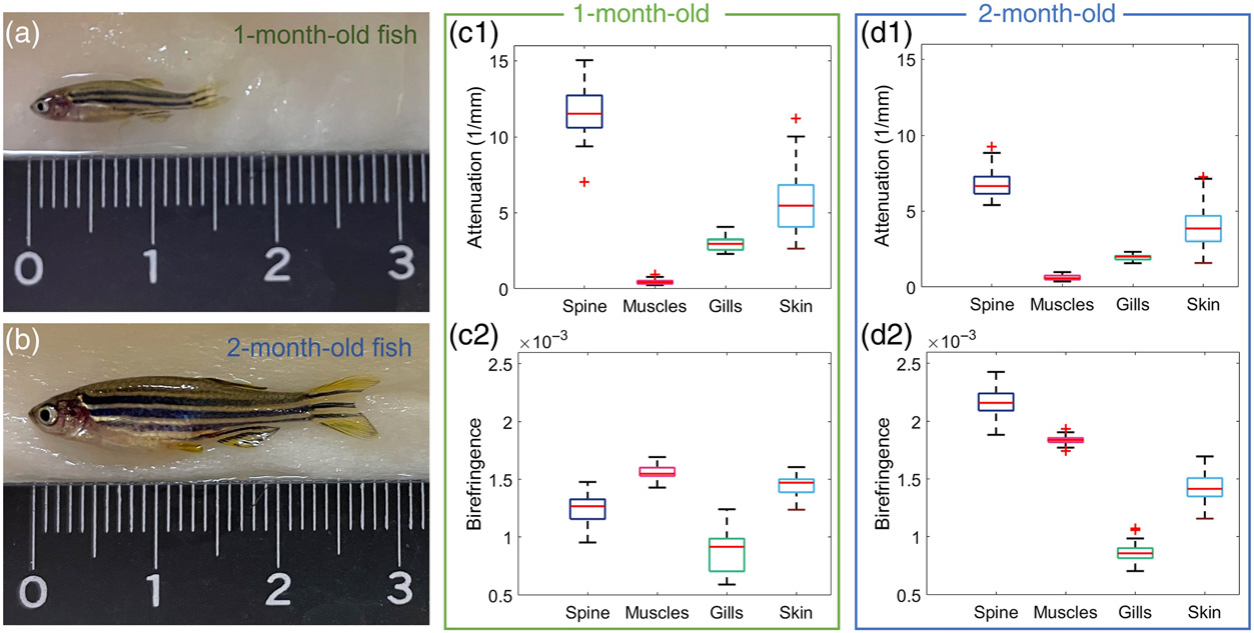
Attenuation and birefringence analysis in various zebrafish organs: (a), (b) white-light photographs of 1- and 2-month-old zebrafish. Box-plot results of the attenuation coefficients and birefringence values in (c1), (c2) 1-month and in (d1), (d2) 2-month-old zebrafish.

A preliminary quantitative analysis of the attenuation coefficients and birefringence signatures for 1- and 2-month-old zebrafish was performed; the quantitative results are presented in Fig. 4 and Table 1. The highest attenuation coefficients, compared with other organs, were observed in the spinal cord region for 1-month-old zebrafish. A decrease of 42% in attenuation was observed in the spinal cord when comparing 1- and 2-month-old animals. The skeletal muscles and gills of both age groups showed low attenuation coefficients. The skin had increased attenuation values with high standard deviations for both age groups. The gills exhibited the lowest birefringence signature both in younger and older zebrafish in comparison with other organs analyzed. The skin showed increased birefringence values compared with the gills. The muscles showed higher birefringence in older animals. In 1-month-old zebrafish, a moderate birefringence was observed in the spine in comparison with the 2-month-old fish, which exhibited increased birefringence values.

**Table 1:** Quantitative evaluation of the attenuation and birefringence (mean value ± standard deviation).

	One-month-old zebrafish		Two-month-old zebrafish	
	Attenuation (${\mathrm{mm}}^{-1}$)	Birefringence	Attenuation (${\mathrm{mm}}^{-1}$)	Birefringence
Spine	11.6±1.6	$1.2\times 10^{-3}\pm 1.2\times 10^{-4}$	6.8±0.8	$2.2\times 10^{-3}\pm 1.1\times 10^{-4}$
Muscles	0.5±0.2	$1.6\times 10^{-3}\pm 0.6\times 10^{-4}$	0.6±0.2	$1.8\times 10^{-3}\pm 0.3\times 10^{-4}$
Gills	2.9±0.5	$0.9\times 10^{-3}\pm 1.7\times 10^{-4}$	2.0±0.2	$0.9\times 10^{-3}\pm 0.9\times 10^{-4}$
Skin	5.7±2.1	$1.5\times 10^{-3}\pm 0.8\times 10^{-4}$	4.0±1.2	$1.4\times 10^{-3}\pm 1.2\times 10^{-4}$

## 4 Discussion

A polarization-sensitive JM-OCT prototype was utilized to perform live imaging in zebrafish in three development stages. Eight-days-, 1-month-, and 2-month-old wildtype animals were examined in a label-free and non-invasive manner. The size of the body of the larvae up to the juvenile/adult zebrafish varies from around 4 to 25 mm^30^ [see Fig. 1(e) and Figs. 4(a) and 4(b), respectively]. Utilizing our prototype in combination with the motorized translation stage and two scanning lenses, the zebrafish in these different age groups were investigated. For the future, this opens the horizon for longitudinal investigations of *in vivo* zebrafish over various development stages.

Anatomical features such as the eye or the gills observed with our JM-OCT prototype are comparable to conventional histology.^31^ Histology provides higher image resolution; however, the JM-OCT results are obtained in a non-destructive and label-free way, with no tissue fixation, sectioning, or staining needed. Also in comparison with histology, the measurements can be performed in real time as volumes are acquired in 6.55 s, which enables live imaging. In addition, JM-OCT is a 3D technique, which is especially of interest when investigating complex 3D structures such as blood vessels. Here the functional extension of OCT-A allows for specifically visualizing blood flow in young and juvenile zebrafish [see Figs. 1(f) and 2(c)]. Using OCT-A data, we are planning to evaluate parameters such as the vessel diameter and blood flow to quantitatively characterize the vasculature. This will be of particular interest as we are currently working on a longitudinal study to investigate tumor growth in a xenograft zebrafish model. The literature has shown the importance of OCT-A in various animal models in observing changes in the vasculature as a biomarker for tumor growth.^32^

It is known that the measured birefringence in PS-OCT is sensitive to fibrous structures.^20^^,^^33^ The literature shows that already at the age of 5-days postfertilization, the smooth muscle layer of the swim-bladder wall is formed.^34^ This musculature layer was identified in the JM-OCT images of the 8-day-old zebrafish by its high birefringence [see Fig. 1(b2)]. The skeletal musculature in 1- and 2-month-old fish [Figs. 2(b1)–2(b2) and Figs. 3(c1)–3(c2)] exhibited a low scattering profile with increased birefringence. The quantitative analysis revealed an increase in birefringence in these two age groups, which might be explained by a growth of these structures.^35^

Furthermore, the eyes in the 8-day-old fish stand out by increased scattering and birefringence [see Figs. 1(a2) and 1(b2), respectively]. In zebrafish as early as 16 to 20 h postfertilization, the highly pigmented RPE is developed.^36^ In the juvenile and young adult zebrafish, anatomical details of the eye were visualized [Fig. 2 and Videos 1 and 2]. The retina, as observed in human and murine eyes, is polarization preserving, whereas the iris and RPE/choroid complex introduce high depolarization; our results are consistent with previous studies.^18^

The vertebral column of the zebrafish consists of the vertebral bodies and spinal cord, which contains a dense network of white fiber tract that densifies with age.^31^^,^^37^ The literature has shown that highly myelinated structures introduce increased birefringence.^38^ The quantitative analysis (Fig. 4) showed increased birefringence in the spinal cord when comparing 1- and 2-month-old zebrafish.

The gills are composed of bony and cartilaginous gill arches that extend into epithelium rich gill filaments.^39^ Low scattering and birefringence were found in the gill filaments with increased values in the arches [see Figs. 3(d1) and 3(d2), respectively]. Similar findings of low scattering and birefringence in the gill filaments have been described in the literature.^20^ Further, the inhomogeneous composition is reflected in the quantitative analysis (Fig. 4) by large standard deviations in attenuation and birefringence values.

The zebrafish skin consists of several layers, namely the dermis containing the bony scales, the hypodermis in which the pigmentation cells are located, and the muscle region underneath.^40^ Dense pigmentation has shown to introduce high scattering and birefringence.^18^ This may be caused by polarization scrambling, which has shown to introduce high retardation. Further, the literature suggests that the dermis exhibits low scattering and birefringence.^20^^,^^28^ The mixture of regions of high and low scattering and birefringence in the fish skin were found in increased standard deviation values (Fig. 4).

In the future, we plan to establish an automatic segmentation of the zebrafish organs based on the scattering and polarization characteristics described in this work. A high imaging speed of 50 kHz in combination with a automatic moving translation stage could be used for high throughput studies. Therefore, the presented JM-OCT prototype offers a flexible and fast tool for zebrafish-based research over a large range of development stages.

## 5 Conclusion

We utilized our JM-OCT prototype to image live wildtype zebrafish in three development stages. The scattering and polarization signatures of various organs were investigated. The presented work showed the potential of JM-OCT as a non-invasive and label-free tool for *in vivo* investigations of zebrafish. Taking all of this into account, we are planning a longitudinal, non-invasive study to investigate tumor growth in a zebrafish model utilizing OCT-A in combination with our JM-OCT prototype.

## Code, Data, and Materials Availability

Data underlying the results presented in this paper are not publicly available at this time but may be obtained from the authors upon reasonable request.
