# Supplementary material for: Multicontrast investigation of in vivo wildtype zebrafish in three development stages using polarization-sensitive optical coherence tomography
Source: J Biomed Opt. 2022 Jan 21;27(1):016001. doi: 10.1117/1.JBO.27.1.016001 (PMC8781523; doi:10.1117/1.JBO.27.1.016001)
Supplement: Supplementary file 3 [file JBO_027_016001_SD003.pdf]

Supplementary material: Multicontrast investigation of in vivo wildtype zebrafish in three development stages using polarization-sensitive optical coherence tomography

Antonia Lichtenegger[1,2,*], Pradipta Mukherjee[2], Junya Tamaoki[3], Lixuan Bian[3], Lida Zhu[2], Ibrahim Abd El-Sadek[2,4], Shuichi Makita[2], Konrad Leskovar[1], Makoto Kobayashi[3], Bernhard Baumann[1], Yoshiaki Yasuno[2]

[1]Center for Medical Physics and Biomedical Engineering, Medical University of Vienna, Austria,
[2]Computational Optics Group, University of Tsukuba, Japan,
[3]Department of Molecular and Developmental Biology, Faculty of Medicine, University of Tsukuba, Japan,
[4]Department of Physics, Faculty of Science, Damietta University, Egypt

1. Jones-matrix optical coherence tomography (JM-OCT)

During the past decades, a great variety of polarization-sensitive optical coherence tomography (PS-OCT) layouts have been introduced. They can roughly be divided into setups with a single circular input state and PS-OCT systems based on sample illumination by multiple polarization states. Jones-matrix OCT (JM-OCT) is based on the illumination by multiple polarization states which is characterized by its ability to measure the three-dimensional distribution of the Jones matrix of the sample. By processing the Jones matrixes, depth-localized phase retardation, which is proportional to birefringence, and local randomness of polarization can be obtained, namely the degree of polarization uniformity (DOPU) [17,18].

2. The JM-OCT setup

Figure 1 (a) shows a sketch of the utilized JM-OCT prototype, reprinted from Li *et al.* [28]. The custom-built JM-OCT setup, which was utilized to investigate the zebrafish, was based on a passive-polarization-delay-based PS-OCT scheme, in which a polarization-delayer (PPD) [yellow box in Fig. 1(a)] multiplexes two incident polarization states in two depths in the OCT imaging field. Besides the PPD, the interference signals are detected by a polarization diversity detection unit (PDD). The PDD splits the interference light signal into two orthogonal polarizations and two OCT signals corresponding to the two-output polarization states measured. Each OCT signal consists of two OCT images in two depths, which are multiplexed by the PPD and correspond to the two incident polarization states. In total four OCT images are obtained. These four OCT images correspond to each combination of the two incident and two output polarization states, and hence form a Jones matrix. From which the polarization properties such as the birefringence and the DOPU can be computed [28].

In Fig. 1 (b) a photograph of the sample arm is shown. The investigated zebrafish are placed in a petri dish onto the motorized stage under the objective lens for imaging.

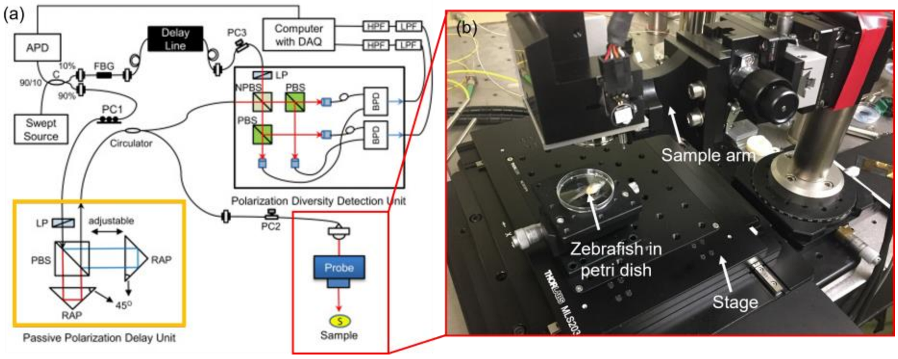

Fig. 1 The Jones-matrix OCT prototype. (a) Sketch of the JM-OCT setup [28]. (APD - amplified photodetector, C – coupler, PC1, PC2 and PC3 - polarization controllers, LP - linear polarizer, PBS - polarizing beam splitter, RAP - right angle prism, S – sample, NPBS - non-polarization beam splitter, BPD - balanced photodetector, LPF - low pass filter, HPF - high pass filter). The passive polarization delay unit is indicated by a yellow rectangle. (b) White-light photograph of the JM-OCT sample arm.

3. JM-OCT data processing

Multicontrast evaluation was performed with the acquired JM-OCT data, including the analysis of the scattering, namely the backscattered intensity and the attention coefficients and the polarization properties namely the birefringence and the DOPU. To generate OCT intensity data (backscattered intensity), the absolute-squared intensities of the four Jones matrix entries, corresponding to the four polarization channels, were averaged [28]. The three-dimensional tissue morphology can be assessed using the intensity-based data. Based on the linear scaled intensity data the attenuation coefficients were calculated using the depth-resolved method devised by Vermeer *et al.* [24]. The attenuation represents a quantitative measurement of the scattering and absorption properties of the investigated tissue.

The depth-resolved birefringence data, also known as the local retardation, were obtained by a local Jones matrix analysis in combination with a maximum *a posteriori* birefringence estimator [25, 26]. Birefringence is a marker for tissue containing highly fibrous structures, such as muscles and fibrosis [18]. JM-OCT can examine the randomization of polarization states among neighboring speckles by using the so-called degree of polarization uniformity (DOPU). Depolarization can be introduced by three main factors, multiple scattering, which can for example be observed in deeper tissue regions, scattering on non-spherical particles, such as melanin, or through surface roughness [27].

Furthermore, OCT angiography (OCT-A) images were obtained by a complex correlation-based approach utilizing four repeated frames. Using OCT-A the vasculature can be investigated in a label-free and non-destructive way. [28]
